# Exposure to an extreme environment comes at a sensorimotor cost

**DOI:** 10.1038/s41526-018-0051-2

**Published:** 2018-09-05

**Authors:** Kyoung Jae Kim, Yoav Gimmon, Sharmeen Sorathia, Kara H. Beaton, Michael C. Schubert

**Affiliations:** 1 Department of Physical Therapy, University of Miami Miller School of Medicine, Coral Gables, FL USA; 2 Neil Spielholz Functional Outcomes Research & Evaluation Center, University of Miami, Coral Gables, FL USA; 3 Laboratory of Vestibular NeuroAdaptation, Department of Otolaryngology-Head and Neck Surgery, Johns Hopkins University School of Medicine, Baltimore, MD USA; 4 Department of Physical Medicine and Rehabilitation, Johns Hopkins University, Baltimore, MD USA

## Abstract

Long duration space flight is known to induce severe modifications in the sensorimotor and musculoskeletal systems. While in-flight strategies including physical fitness have been used to prevent the loss of bone and muscle mass using appropriate rehabilitative countermeasures, less attention has been put forth in the design of technologies that can quickly and effectively assess sensorimotor function during missions in space. The aims of the present study were therefore (1) to develop a Portable Sensorimotor Assessment Platform (PSAP) to enable a crewmember to independently and quickly assess his/her sensorimotor function during the NASA’s Extreme Environment Mission Operations (NEEMO) and (2) to investigate changes in performance of static posture, tandem gait, and lower limb ataxia due to exposure in an extreme environment. Our data reveal that measuring the degree of upper body balance and gait regularity during tandem walking using PSAP provided a sensitive and objective quantification of body movement abnormalities due to changes in sensorimotor performance over the duration of mission exposure.

## Diagnostics: New test for sensory and motor problems

An assessment tool that combines body sensors and a walking task can detect functional abnormalities following prolonged exposure to an extreme environment. Michael Schubert from Johns Hopkins University in Baltimore, MD, USA, and colleagues integrated five wireless inertial sensors with a computer tablet to develop what they call the Portable Sensorimotor Assessment Platform (PSAP). They tested the platform on crewmembers living in an undersea laboratory, assigning various physical tasks to investigate posture, gait, and limb function. They found that using PSAP while participants walked in a straight line with one foot immediately in front of the other—heel to toe—offered an accurate and unbiased picture of upper body balance and gait regularity. Similar methods could help measure sensorimotor function and avoid injury among astronauts on extended missions to Mars or elsewhere.

## Introduction

NASA intends to send humans to Mars in the 2030s.^1,2^ This will involve long duration exposure to conditions of microgravity that may impair the crewmembers' sensorimotor function when re-exposed to gravitational environments. Different studies on human adaptation to space flight reveal a clear and serious disruption of sensorimotor function,^3,4^ including postural and oculomotor control systems.^5–10^ Astronauts returning to a 1G environment from both short (8–15 days) and long (>160 days) duration space flight report difficulty with locomotion upon return to Earth to include ataxic gait, a tendency to fall to the outside when turning corners,^11^ a cautious gait that involves keeping the arms raised as if to prevent a fall, and use of a widened base of support with reduced step frequency.^12,13^ Many have a reluctance to move their head.^5^ In response to these postural and gait problems, NASA scientists have developed a clever obstacle course called the functional mobility task in which astronauts are asked to maneuver around and over objects, all while walking on an unsteady foam surface.^10,14^ Studies reveal astronauts require a median 48% increase in time to complete the obstacle course upon return to Earth. Other studies reveal a decreased coordination between the head and trunk during post-flight locomotion, which tends to be worse in naive flyers.^6^

**Fig. 1:**
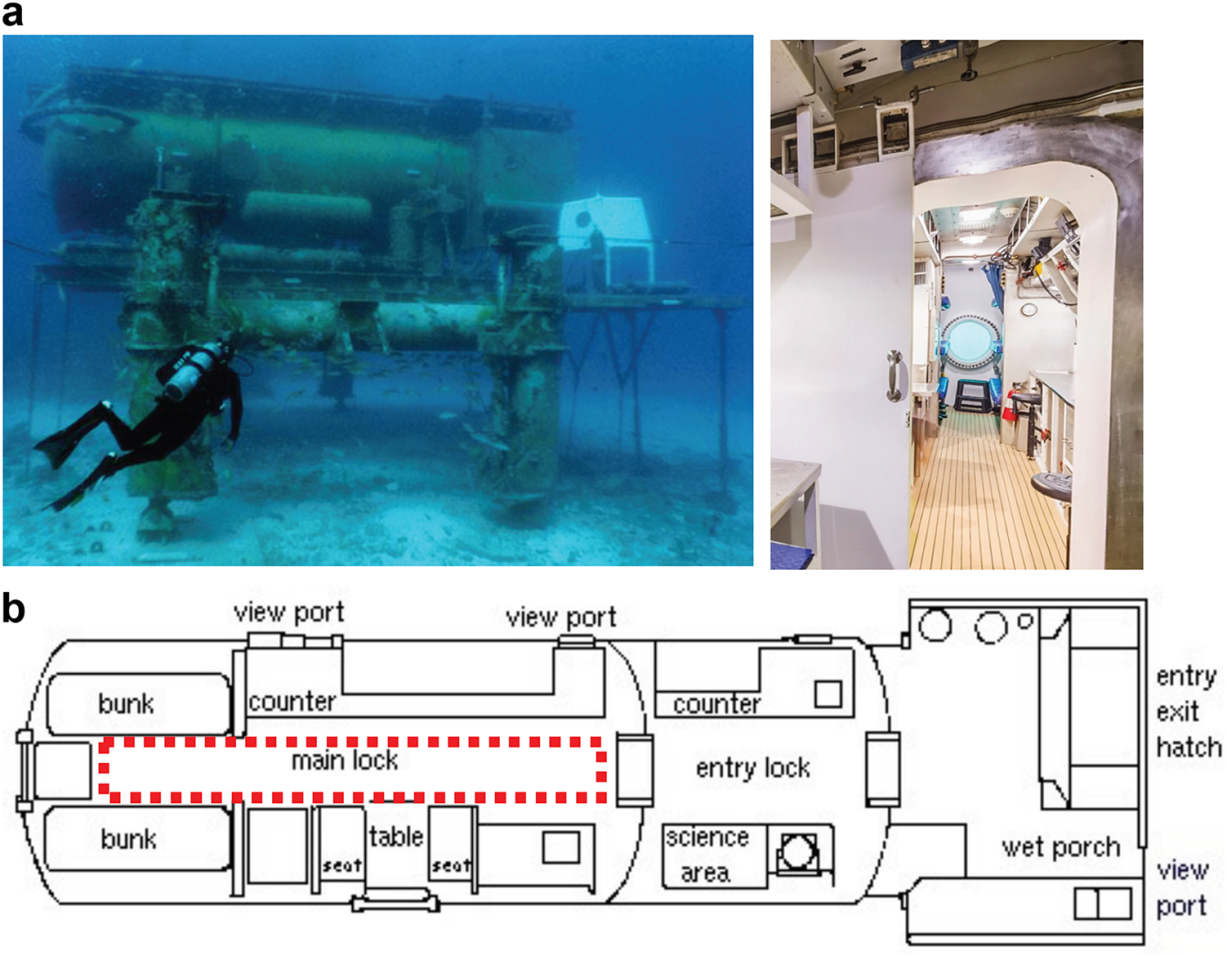
**a** Aquarius Reef Base seen from the outside (left) and main lock seen from the entry lock (right) and **b** Aquarius’ floor plan. The dotted red line indicates where the crewmembers performed the PSAP testing

NASA employs various research analogs that mimic some of the physical, psychological, and emotional challenges that crewmembers will face to study and prepare for future exploration missions. The NASA Extreme Environment Mission Operations (NEEMO) project is one such research analog.^15^ During NEEMO missions, individuals live for up to 3 weeks inside the Aquarius habitat, the world’s only undersea research station, which is located 19 m undersea and 5.6 km off the coast of Key Largo in the Florida Keys National Marine Sanctuary (Fig. 1).

NEEMO crewmembers, known as aquanauts, experience some of the same challenges in Aquarius that they would on a distant asteroid, planet, or moon. Some of these challenges include living at a saturated atmospheric pressure (~2.5 atm.), limited space to move, and the persistent threat of danger. Therefore, the Aquarius habitat mimics some of the isolation, extreme conditions, and genuinely alien environment of space, thus making it relevant to studying human behavior relevant for space exploration. Additionally, any sensorimotor challenges identified while living in Aquarius may have implications for the aquanaut training activities within the duration of the NEEMO mission.

**Fig. 2:**
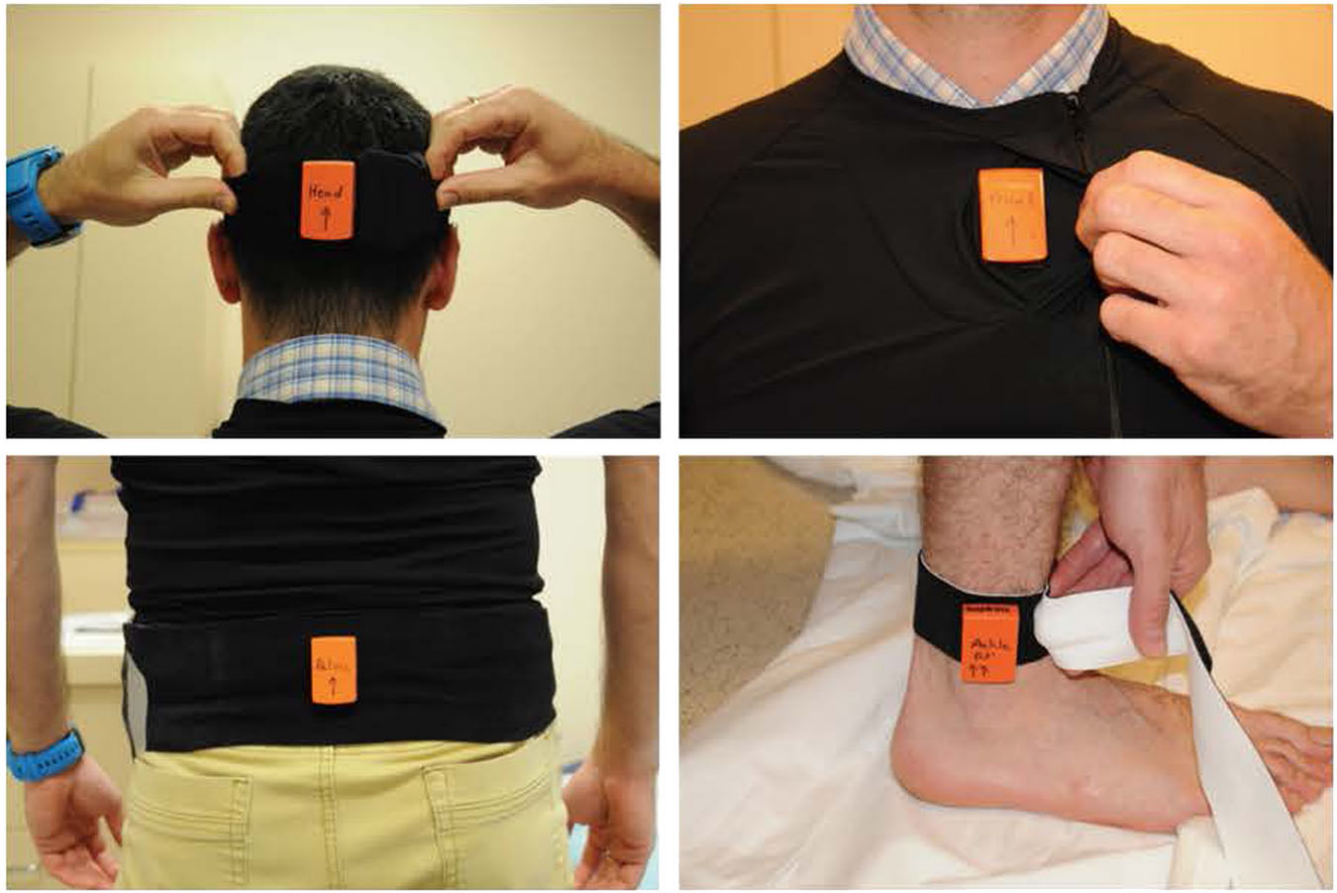
We used five MTw Xsens sensor units to record kinematic data. Each unit comprises an accelerometer, gyroscope, and magnetometer. Five sensors were placed including the head, trunk, pelvis, left and right ankles

**Fig. 3:**
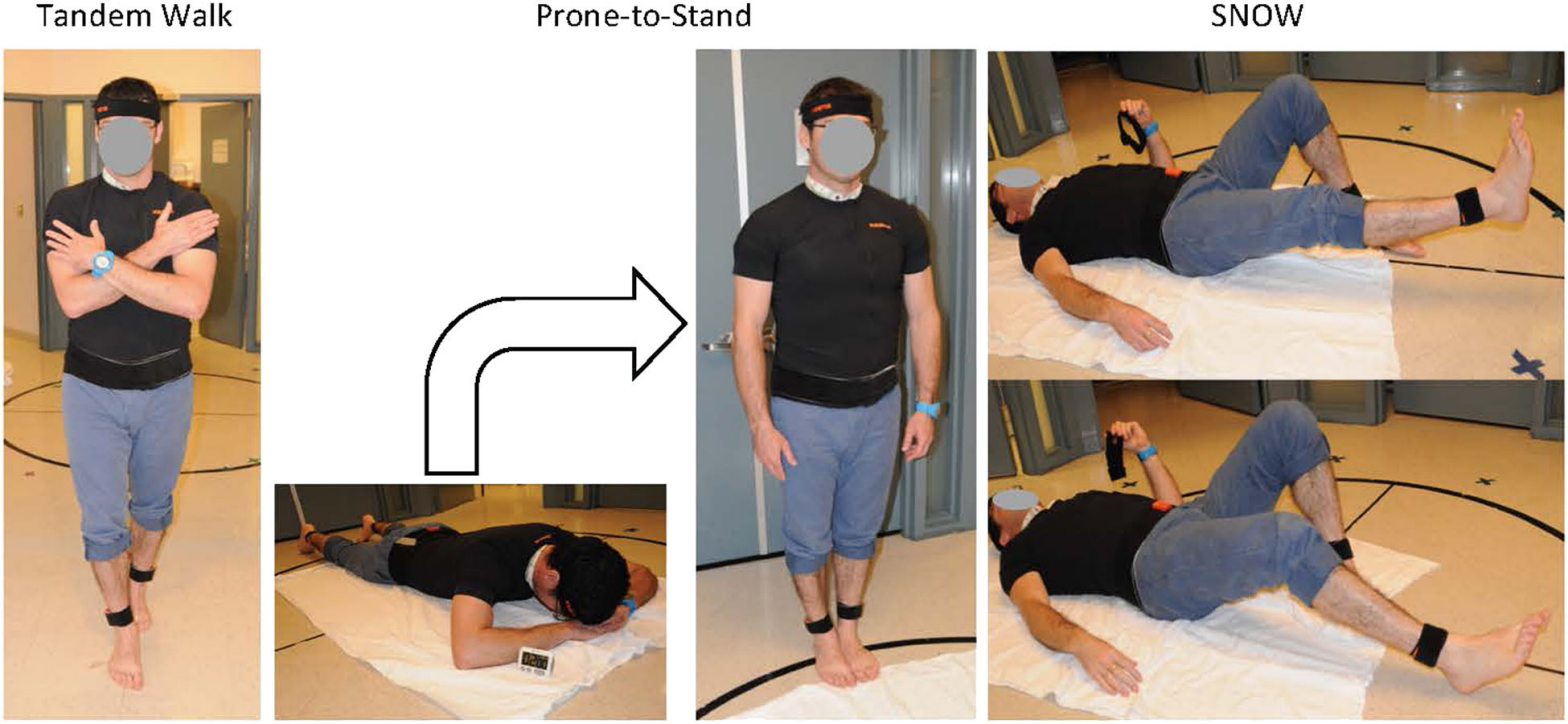
PSAP test battery included the Tandem Walk test, Prone-to-Stand test, and SNOW test. Each test included eyes open and eyes closed conditions. For SNOW testing, two additional conditions involved heel in the air and on the ground

Given the duration of space travel to Mars, it would be advantageous to design a technology that can quickly and effectively assess sensorimotor function on a periodic basis. Once identified, the crewmembers may then be able to quickly nullify related symptoms using appropriate individualized rehabilitative countermeasures. Accordingly, we developed the Portable Sensorimotor Assessment Platform (PSAP) to enable a single crewmember to independently assess his/her sensorimotor function during the NEEMO 21 and 22 missions. PSAP is based on five body-worn inertial sensors (Fig. 2) integrated with a handheld computer tablet. The purpose of this study was to investigate how well PSAP would monitor any change in sensorimotor performance associated with exposure to the NEEMO environment. Sessions consisted of three tests: (1) Tandem Walk test, (2) Prone-to-Stand, and (3) “S” “N” “O” “W” letter leg-writing test (Fig. 3). Each test was done with eyes open (EO) and again with eyes closed (EC). We hypothesized that exposure to the NEEMO extreme environment would lead to a change in inertial sensor-based measures of tandem gait, static posture, and lower limb ataxia as measured with the PSAP system—all as an indicator of reduced sensorimotor control.

## Results

### NEEMO crewmembers

**Fig. 4:**
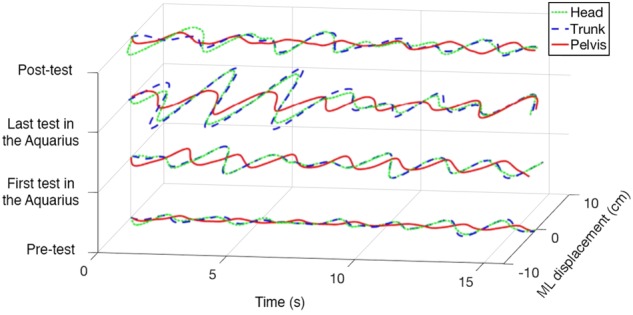
An example of the ML displacement curves in a crewmember during pre-, intra-, and post-missions of the tandem walk test in the EC condition. The dotted green line, dashed blue line, and solid red line are the ML displacement curves observed from the head, trunk, and pelvis sensors, respectively

**Fig. 5:**
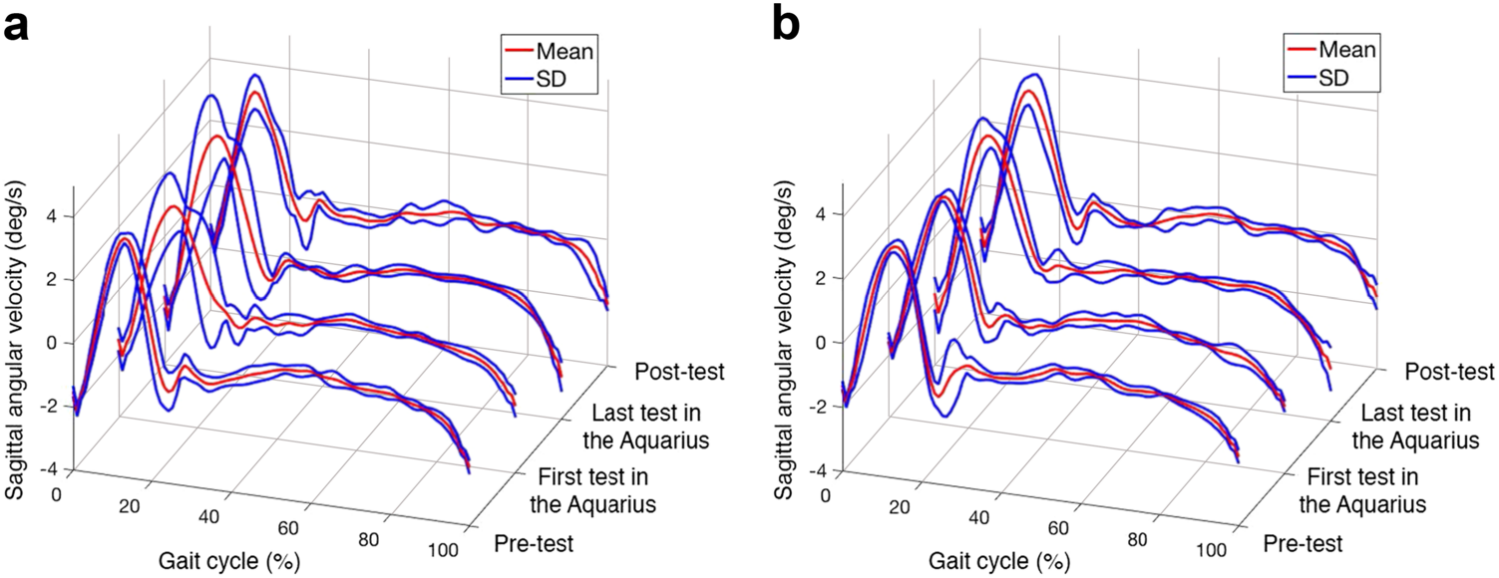
An example of variation of stride patterns observed over time from the **a** right and **b** left ankle sensors in a crewmember during pre-, intra-, and post-missions of the tandem walk test in the EO condition. The red and blue lines are the mean (stride template) and one standard deviation (SD) of segmented gait strides, respectively

We found statistically significant changes in parameters related to tandem walking in both the EO and EC conditions, Table 1. Figure 4 presents an example of the medio-lateral (ML) displacement curves observed at the head, trunk, and pelvis sensors in a crewmember during pre-, intra-, and post-missions of the tandem walk test in the EC condition. Figure 5 illustrates an example of the variability of stride patterns over time that were observed from the right and left ankle sensors in a crewmember during the pre-, intra-, and post-missions of the tandem walk test in the EO condition.

**Table 1:** Mean and standard deviation (SD) of tandem walk variables for all crewmembers during EO and EC conditions

Condition	Variable	Pre-test	First test in the Aquarius	Last test in the Aquarius	Post-test
EO	Displacement Area-Head	28.68 (9.92)	21.46 (6.36)	31.87 (13.95)	29.43 (5.42)
	Displacement Area-Trunk	32.08 (6.62)	27.08 (7.85)	35.29 (12.35)	32.74 (11.89)
	Displacement Area-Pelvis	19.95 (7.84)	22.87 (17.82)	18.09 (9.11)	21.36 (16.22)
	Gait Regularity-Right Mean	1.29 (0.29)	1.49 (0.49)	1.29 (0.51)	1.65 (1.01)
	Gait Regularity-Right SD	0.35 (0.11)	0.73 (0.29)	0.52 (0.20)	0.78 (0.52)
	Gait Regularity-Right Range	0.91 (0.27)	1.78 (0.72)	1.34 (0.57)	1.93 (1.35)
	Gait Regularity-Left Mean	1.30 (0.38)	1.56 (0.52)	1.27 (0.33)	1.39 (0.65)
	*Gait Regularity-Left SD* ^e^	0.69 (0.31)^a^	0.61 (0.27)	0.47 (0.34)	0.54 (0.28)
	*Gait Regularity-Left Range* ^e^	1.73 (0.73)^a^	1.49 (0.69)^c^	1.12 (0.88)	1.25 (0.75)
EC	Displacement Area-Head	37.85 (6.42)	37.89 (8.06)	50.19 (13.26)	65.15 (22.10)
	*Displacement Area-Trunk* ^e^	47.13 (19.88)^b^	54.07 (14.44)^d^	76.21 (16.95)	91.95 (21.34)
	Displacement Area-Pelvis	27.25 (12.44)	30.22 (13.04)	21.14 (5.13)	28.46 (8.90)
	*Gait Regularity-Right Mean* ^e^	2.84 (1.25)	7.70 (9.12)	15.45 (13.45)	12.61 (9.45)
	*Gait Regularity-Right SD* ^e^	1.31 (0.85)	6.34 (8.91)	13.11 (14.83)	7.11 (5.33)
	*Gait Regularity-Right Range* ^e^	3.40 (2.30)	14.70 (20.18)	30.88 (33.16)	16.54 (12.15)
	Gait Regularity-Left Mean	2.91 (1.68)	9.51 (8.25)	8.61 (7.98)	10.18 (11.55)
	Gait Regularity-Left SD	2.08 (1.74)	6.93 (7.66)	5.13 (7.38)	7.62 (12.01)
	Gait Regularity-Left Range	4.65 (3.48)	15.30 (15.22)	12.15 (17.44)	16.15 (23.83)

The mean displacement area (cm*s) is presented for head, trunk, and pelvis for the duration of the tandem walk. Larger displacement indicates larger sway, a deterioration of balance. Gait regularity (deg/s) evaluates the similarity of consecutive strides during the tandem walk and is the difference between the template stride and each stride. Larger Regularity indicates a larger distance from the template stride (less similarity), a deterioration of balance

^a^Significant difference between pre-test to last test under the water

^b^Significant difference between pre-test to post-test

^c^Significant difference between 1st test to last test under the water

^d^Significant difference between 1st test under the water to post-test

^e^Significant correlation to time spent under the water

During the EO tandem walk condition, both variability (*r* = −0.477; *p* = 0.021) and range (*r* = −0.491; *p* = 0.017) of the Gait Regularity for Left steps were inversely correlated with duration of time spent by the crewmembers inside the habitat. These two variables (variability and range) for left steps show a similar pattern of progressive reduction (*p* = 0.003) as long as crewmembers were inside the habitat. Once emerged from the habitat, each measure increased but was still lower than pre-test values. Repeated measures ANOVA revealed a significant difference (*p* = 0.003 for variability; *p* < 0.001 for range) between the pre-test and the last day of the mission under the water (Table 1). Gait regularity during rightward steps was not significantly different between the repeated measures, however, a large variability in performance was noted (Table 1). Displacement variables measured from the head, trunk, or pelvis were not significant in crewmembers over the duration spent inside the habitat for EO tandem walking. There were no significant correlations for any of the variables examined for the Prone-to-Stand or the SNOW test during EO.

During the EC tandem walk condition, we found significant correlations between the mission duration and gait regularity as well as trunk displacement. In particular, mean (*r* = 0.524; *p* = 0.010), standard deviation (*r* = 0.436; *p* = 0.038), and range (*r* = 0.448; *p* = 0.032) of gait regularity for rightward steps EC were all positively correlated with the duration the crewmembers spent inside the NEEMO habitat (Table 1). However, these positively correlated gait variables were not significantly different over time based on the repeated measures ANOVA. Displacement Area-Trunk EC (*r* = 0.532; *p* = 0.009) was both significantly correlated and significantly increased over the duration of the mission comparing pre-test and first measurement under the water with the post-test and final measurement under the water (Table 1). There were no significant correlations for any of the variables examined for the Prone-to-Stand or the SNOW test during EC.

### Control subjects

There were no significant correlations or significant differences for any of the variables examined with the control subjects (Table 2).

**Table 2:** Mean and SD of tandem walk variables for all control subjects during EO and EC conditions

Condition	Variable	1st Measurement	2nd Measurement	3rd Measurement	4th Measurement
EO	Displacement Area-Head	28.02 (14.56)	27.57 (22.46)	28.88 (31.38)	31.26 (17.74)
	Displacement Area-Trunk	28.14 (13.51)	24.82 (16.52)	26.94 (26.82)	26.22 (15.09)
	Displacement Area-Pelvis	17.75 (9.08)	16.42 (13.06)	11.98 (6.23)	14.42 (7.03)
	Gait Regularity-Right Mean	1.61 (0.35)	2.22 (0.82)	1.87 (0.71)	2.80 (2.83)
	Gait Regularity-Right SD	0.52 (0.22)	0.51 (0.29)	0.69 (0.36)	1.71 (3.18)
	Gait Regularity-Right Range	1.24 (0.49)	1.18 (0.71)	1.61 (0.82)	3.72 (6.84)
	Gait Regularity-Left Mean	2.17 (0.70)	1.96 (0.49)	1.74 (0.42)	1.93 (1.15)
	Gait Regularity-Left SD	0.71 (0.29)	0.75 (0.29)	0.56 (0.34)	0.96 (0.78)
	Gait Regularity-Left Range	1.55 (0.64)	1.77 (0.70)	1.30 (0.81)	2.18 (1.97)
EC	Displacement Area-Head	62.34 (52.23)	37.73 (32.95)	47.29 (44.53)	53.24 (55.51)
	Displacement Area-Trunk	68.29 (50.65)	40.32 (30.60)	45.34 (43.42)	54.26 (51.75)
	Displacement Area-Pelvis	20.75 (7.81)	16.48 (9.82)	14.27 (9.68)	19.35 (7.27)
	Gait Regularity-Right Mean	5.69 (4.98)	6.18 (6.74)	7.83 (4.96)	5.92 (4.54)
	Gait Regularity-Right SD	2.41 (2.81)	2.37 (3.39)	3.83 (4.06)	3.07 (4.20)
	Gait Regularity-Right Range	5.48 (6.31)	5.90 (7.61)	8.44 (8.68)	6.67 (9.16)
	Gait Regularity-Left Mean	6.41 (5.18)	4.59 (2.54)	7.03 (8.29)	8.77 (8.30)
	Gait Regularity-Left SD	3.11 (2.47)	2.05 (1.25)	1.33 (1.19)	3.90 (3.28)
	Gait Regularity-Left Range	7.06 (5.65)	4.18 (2.57)	3.11 (2.87)	8.09 (6.27)

No significant correlations for any of the variables with the time, nor any significant differences between the measurements

## Discussion

The results of our study revealed that the instrumented tandem walk test within the PSAP suite of measures is useful for identifying decreased ability to tandem walk over the duration that crewmembers participated in one of NASA’s extreme environment research analogs (i.e., NEEMO). First, using three inertial sensors positioned on the upper body, we showed changes in the ML displacement of the trunk and to a lesser extent, the head and pelvis during tandem walking in the EC condition. In our observation with PSAP, crewmembers maintained pelvis stability (estimated by the pelvis inertial sensor) to protect the center of mass position and prevent a fall. In contrast, the trunk segment showed a significant ML displacement area over time while the head displacement trended towards significance (Fig. 4). This result is best explained recognizing the important role the upper body (trunk and neck) has in compensating for the movement constraints of the lower limbs as occurs during tandem walking. The tandem walk task constrains the lower body and forces increased motion variability from the upper body to prevent a fall. Our pelvic, trunk, and head sensor data, from the crewmembers (Table 1) as well as from the control subjects (Table 2), supported this compensatory response.

It is well known that long-duration spaceflight negatively affects the human body. For example, the loss of bone and muscle mass are the most apparent, detrimental effects of microgravity.^16,17^ Sensorimotor function too, is altered by exposure to the microgravity environment of space, resulting in atony, atrophy, decreased gait speed, and reduced muscle power capabilities.^18,19^ To prepare astronauts for working in space, the Johnson Space Center (JSC) uses the Neutral Buoyancy Laboratory (NBL) as a training analog for space’s unique microgravity environment.^20^ Our data suggest crewmembers exposed to the duration of the NEEMO mission suffer a sensorimotor cost. This cost may be avoidable, or adaptable given the plasticity existent in sensorimotor function. We are unaware of how training in the NBL might similarly impact sensorimotor control as we have illustrated occurs in crewmembers participating in the NEEMO environment. Certainly, the NEEMO mission is more challenging than training for a single day in the NBL, not to mention its isolated and extreme environment that may also have a negative impact. It would be interesting however, to measure tandem walk before and after training in the NBL, to see if our results can be replicated. While this may seem unlikely given the differences in both duration and atmosphere (i.e., depth) between NEEMO and the NBL, we did show reduced balance at the early stage of the NEEMO mission—that worsened with time under water. Additionally, tandem walk did not completely recover until the crew returned to land, with trunk displacement still being significantly abnormal at the time of final measurement on land. This is concerning and suggests that the altered pressure environment in combination with many other factors experienced by the crew during a NEEMO mission (e.g., confined space, participation in extravehicular activities (EVAs), possible changes in sleep patterns, higher stress due to intense mission days) leads to sensorimotor changes similar to those experienced during spaceflight.

It has been suggested that the contribution of proprioceptive information from the lower limbs to maintaining posture might worsen during space flight due to the prolonged weightlessness.^21^ The tonic vibration reflexes have been shown to be diminished in the micro-gravitational phase of parabolic flight and are increased during the hyper-gravitational phase of parabolic flights.^22,23^ This suggests that the proprioceptive sensors are diminished in reduced gravity. In our study, it remains possible that a proprioceptive deficit contributed to the poor balance performance based on a deprived sensory input from performing atypical tasks such as participating in EVA up to 4 h per day (i.e., reduced time spent in weight bearing). We did not explicitly measure proprioception; and we are unaware of studies comparing the relationship between weight bearing activity and proprioception in a 1G environment. However, if an inverse relationship between weight bearing and proprioception exists, then the central nervous system (CNS) would have an impaired sensory reweighting process to maintain postural control. The healthy CNS integrates continuous afferent information from the vestibular, visual, and proprioceptive systems.^24^ Sensory redundancy enables the CNS to select the most reliable afference for the most efficient postural control (called sensory reweighting), with a selection priority that adapts to the demands of the postural task. In our study, in order to maintain balance during tandem walking with EO, the CNS would “reweight” reliance to the visual system as a compensatory strategy given the altered somatosensory input. Indeed, we found this condition to be abnormal. Furthermore, the tandem walk task worsened with EC—which may reflect a doubly impaired sensory afference (absent vision and the putative proprioceptive impairment). Finally, if such proprioceptive deficits are progressive, then balance would continue to degrade, which our data also supports (Table 1).

Next, we also described the relationship between gait regularity and changes in sensorimotor performance during the tandem walk test. From the two inertial sensors positioned on the left and right ankles, we showed that the regularity of tandem gait deteriorates over the duration of the NEEMO mission, as we hypothesized. This contrasts with our control data that showed no change in regularity of tandem gait over time (Table 2). Specifically, our data revealed a difference in Gait Regularity between right and left steps in the EO or EC tandem walking conditions. During EC tandem walking, Gait Regularity for Right steps was correlated with and progressively worse over the mission duration. This suggests worsening postural stability for the rightward steps. However, during EO tandem walking, variables of the Gait Regularity for Right steps were neither correlated with mission duration nor different over time. Only the Gait Regularity for Left steps inversely correlated with duration of time spent by the crewmembers inside the habitat, suggesting improved postural stability over mission duration. We have considered whether limb dominance might be responsible for the apparent discrepancy in gait regularity behavior of the lower limbs during tandem walking in the EO and EC conditions. Prior literature on gait revealed the dominant limb is more responsible for forward progression while the non-dominant limb provides support and postural stability.^25–27^ In our study, 9 of 10 crewmembers reported right limb dominance. If the prior literature on pattern of “forward limb propulsion” vs. “postural stabilization” persists during tandem walking with EO, then our data supports this observation; crewmembers have improved left step regularity (non-dominant) to ensure dynamic balance (no significant difference in Displacement variables measured from the head, trunk or pelvis) and forward propulsion of the leading right limb (no significant difference in Gait Regularity for right steps). However, during EC tandem walking the overall postural stability from the non-dominant left limb cannot ensure a stable forward progression of the right limb (e.g., step regularity). For this reason, the variables of the Gait Regularity for Right steps (dominant limb) could continue to degrade. The absence of such variability in the control subjects supported this rationale and demonstrates the sensitivity of the gait regularity measure for tandem walk.

The present study involved several limitations. First, our results are limited to the small sample sizes inherent to research involving NASA personnel or extreme environments. Thus, our results should not be generalized beyond the NEEMO environment. Next, although some of the variables we developed to measure gait (i.e., gait regularity) did reveal behavioral change during tandem walk, the use of a “single” variable may not be sensitive to identify the complex behavior of gait. We believe, however, that similar studies examining sensorimotor function in situ over time are now warranted given these results. Future studies would also benefit from explicitly correlating the participation in EVA’s (i.e., “unloading” of body weight) with sensorimotor performance. In concert with measuring sensorimotor function, the inclusion of physiologic measures (i.e., cerebral blood flow velocity) may further clarify how best to develop countermeasures. It remains possible that the unique NEEMO habitat itself influenced some of the decrement in sensorimotor performance that we reported, however this would not explain the progressive decrement that we showed. Finally, we did not find statistically significant correlations for the variables examined during the Prone-to-Stand or SNOW testing, suggesting these non-gait tasks are not as sensitive to identify sensorimotor change in the NEEMO environment. Our data suggest crewmembers involved in long duration research analog missions or space travel (i.e., initiatives to Mars, commercial space travel) be monitored for sensorimotor function using the tandem walk task. NASA has expressed concern with crewmembers’ ability to egress the vehicle in case of an emergency or after long-duration space travel. Therefore, it remains essential to measure sensorimotor function within the functional constructs of gait. We believe PSAP could be useful as both an in-flight and pre/post-flight assessment tool in its current configuration (five inertial sensors and a mobile tablet). Clearly, testing PSAP within a reduced gravity environment remains critical. This reduced gravity environment however is not in itself a limit given crewmembers currently exercise within Node 3 of the International Space Station using treadmills with fixation harnesses. We envision similar methods for assessing fall risk during gait in reduced gravity environments.

## Methods

### Portable Sensorimotor Assessment Platform

PSAP is comprised of a Microsoft Surface Pro 3 tablet computer (Microsoft Corporation, Redmond, WA, USA) and five small MTw inertial sensors with a sampling frequency of 100 Hz that are part of the MTw Software Development Kit (Xsens Technologies BV, Enschede, Netherlands). The MTw inertial sensors were chosen in part based on their established commercial validation estimating human kinematics against the gold-standard optical motion capture systems (https://www.xsens.com/research/). Each sensor comprises three tri-axial devices (accelerometer, gyroscope, and magnetometer) with ranges of ±160 m/s^2^, ±2000 deg/s, and ±1.9 Gauss, respectively. Acceleration, rate of rotation, and the strength of the Earth’s magnetic field along three perpendicular axes were recorded for each unit. Measurements were transmitted over a radio frequency connection (ZigBee) to the Awinda Station connected to the tablet via a USB interface. As shown in Fig. 2, one sensor was secured to the back of the head (midline), the trunk sensor was placed inside the pocket of a form-fitting custom short-sleeve shirt 1 cm to the right of upper part of the sternum. The pelvis sensor was secured on the back at the level of the L2 vertebra above its spinous process. The ankles sensors were attached to the lateral side of the ankle just above the lateral malleolus.

### Experimental setup and data collection

Ten crewmembers (six from the NEEMO 21 mission and four from the NEEMO 22 mission) as well as five control subjects were recruited for this study. All participants were currently healthy performing their testing activities, and did not report any illness, injury, or prior surgery. All subjects were also free from sensorimotor, neuro-vestibular, and musculoskeletal issues. Anthropometric measurements, including height and weight, were self-reported to the study investigators. All crewmembers gave informed consent, as approved by both the Johns Hopkins University and the NASA JSC Institutional Review Boards. Healthy control subjects were consented as approved by Johns Hopkins University. Our central goal was to evaluate any sensorimotor impairments over time of living in an extreme environment. Therefore, data was collected pre-, intra-, and post-missions. Specifically, each of the ten crewmembers participated in both a pre-mission training session and baseline data collection at the JSC. During the mission weeks in Florida, data was collected on mission day 2 (as soon as possible after “splashdown” into the Aquarius habitat), mid-mission, and finally post-mission within a few hours of returning to land. Crewmembers lived inside Aquarius for up to 2 weeks at a time. During both missions, all testing was conducted in the Aquarius habitat main lock (Fig. 1) and recorded via video feeds by a member of the investigator team, which was critical to verify the test being performed and identify any causes for errant kinematic data (i.e., test subject moved out of the way of a crewmember walking by). During all testing, the crewmembers donned MTw sensor units at the head, trunk, pelvis, and both ankles to measure movements of different body segments. Not every crewmember completed each sensorimotor test.

Tandem walk is a straight-line heel-to-toe walking test and quantifies postural stability and locomotor control.^28,29^ Prone-to-Stand is a part of the NASA’s Functional Task Test (FTT) to assess changes in multiple physiological systems including cardiovascular function and sensorimotor function after space flight.^30^ The test quantifies postural stability during potential orthostatic intolerance. The “S” “N” “O” “W” test is a measure of lower limb motor control tasking subjects to write the letters “S” “N” “O” “W” with their legs. We proposed the SNOW test to quantify lower limb ataxic-like symptoms using sensor data. Each of the Tandem Walk, Prone-to-Stand, and SNOW tests were done with EO and with EC. All data recorded during the three tests were automatically saved to the PSAP tablet.

For the EO tandem walk test, the subject began by standing inside the main airlock hatch doorway with arms folded across the chest and one foot in front of the other with heel touching toes. The subject stood still while looking straight ahead, counted to 5, and then walked heel-to-toe approximately 15 ft until he/she reached the bunkroom entrance. The subject then stood still, counted to 5 again, and returned to the start location for their next trial. During each test session, the subject completed three EO and EC trials, respectively. During the EC tandem walk trials, another crewmember guarded the subject while walking. Control subjects repeated the same tandem walk protocol. Controls walked a distance of 15 ft in a hallway at the Johns Hopkins Hospital. Each control repeated the tandem walk on 4 separate days over a period of 8 days using the PSAP system. For the Prone-to-Stand test, the subject lay prone for 2 min and then stood up straight with his/her feet together quietly for 1 min.^30^ During each test session, the subject completed three EO and EC trials, respectively. In the SNOW test, the subject lay supine with one knee bent and foot flat on floor. The straight leg was then raised several inches from the ground and the subject “wrote” the letters S, N, O, W with the raised leg. The subject then “wrote” the same letters with the straight leg resting on the ground while tracing that heel along the floor. For the SNOW test, eight trials were completed: right and left leg in the air with EO, right and left leg in the air with EC, right and left leg on the floor with EO, and right and left leg on the floor with EC. Control subjects did not do Prone-to-Stand test and the SNOW test.

### Data processing

#### Post-processing of sensor data for human motion analysis

A fundamental problem in human motion analysis using inertial sensors is the sensors’ local coordinate axes are not aligned with any physiologically meaningful axis. For example, inertial sensors placed on the back are unlikely to be mounted perfectly to the horizontal and vertical axes due to curvature of the spine. This tilt affects the output of the sensor. Thus, knowledge of the placement of the sensor relative to the cardinal directions of the body is necessary to accurately relate observed motions of the sensor placed on each body segment. To resolve this issue, we virtually rotated the orientation of the head, trunk, and ankle sensors with respect to the pelvic sensor’s reference orientation. We also compensated for non-orthogonality of the sensors using algorithms that removed the gravity component and instead determined the true acceleration measured from that sensor.^31^ These procedures allowed us to use the same axis orientation of the sensors and, thus, to account for physiologic differences between subjects. All data processing was done within MATLAB^®^ R2017b (MathWorks, Inc., MA, USA).

#### Quantification of balance and gait regularity during tandem walking

To determine control of balance during tandem walking, we examined not only trunk movement patterns but also head and pelvis movement patterns to establish ways in which the coordination between these segments contribute to balance of the upper body.^32^ Specifically, we quantified ML displacement of the head, trunk, and pelvis during tandem walking. We estimated each lateral displacement by double integration with high-pass filtering of the resulting acceleration from a tilt-compensated sensor donned at the head, trunk, and pelvis, respectively.^33,34^ The amount a crewmember swayed from side to side during tandem walking was measured by the area under the ML displacement curve using integration.

To assess gait regularity of the lower limbs during tandem walking, we derived gait outcomes from the ankle sensors. Gait regularity is commonly defined as the similarity of consecutive strides.^35,36^ Deterioration of gait regularity over time can be an indicator of progressive sensorimotor deficits.^37^ To detect the slight changes in tandem gait patterns we used dynamic time warping (DTW), which is a commonly used method for computing the similarity between two time sequences.^38^ DTW matches signals by warping the template upon the target signal to provide a distance measure between signals. This gait analysis method enables us to compare test strides to reference strides.^39^ Specifically, we segmented individual strides based on detected toe-off events^40–42^ from the gait sequences (sagittal angular velocities measured at left and right ankle sensors). Next, we calculated stride templates by averaging the segmented right and left strides, respectively. Each consecutive stride was compared to the stride template to determine distances using DTW. Finally, we used the mean, standard deviation (SD), and range of calculated distances as measures of gait regularity. Uncoordinated lower limb movement due to a sensorimotor control problem during tandem gait may lead to irregular lurching of steps, which causes an increase in the gait regularity parameters (including mean, SD, and range).

#### Quantification of standing balance

Body sway findings during standing were quantified by sensors at the head, trunk, and pelvis. We used computations of sensor-traced path and area in the ML and anterior–posterior (AP) directions observed at the head, trunk, and pelvis during standing up after prone as previously described.^43,44^ Sensorimotor deficits may have a detrimental effect on postural control, which might cause an increase in body sway during the Prone-to-Stand test.

#### Quantification of writing the letters S, N, O, W with a leg

Crewmembers who sustain sensorimotor deficits may have symptoms including impaired coordination in the legs, frequent stumbling, unsteady movements, and/or unexpected fatigue during limb movements.^45,46^ Standardized clinical sensorimotor assessments rely on observational and subjective measures of functional task performance. Analysis of data recorded from an ankle inertial sensor could allow the identification of specific functional deficits during limb writing motions (the SNOW test). Specifically, we employed the jerk and sample entropy to quantify such ataxic-like symptoms using sensor data.

We used the square of the magnitude of jerk of the lower limb integrated over the entire S, N, O, W writing motions. Jerk is a measure of smoothness, which is mathematically defined as the rate of change of acceleration.^47^ One of major goals of motor coordination of the lower limb during SNOW is the production of the smoothest possible movement. However, frequent stumbling of the raised leg due to decreased coordination ability might generate a jagged motion trajectory during limb writing. Accordingly, the increased stumbling in crewmembers during mission days may be equated to the larger mean-square jerk than those observed in the baseline data collection. Additionally, we used sample entropy, which is usually used as a measure of complexity for physiological time series. An entropy-based analysis, such as sample entropy, evaluates the appearance of repetitive patterns of a time series to quantify the degree of its regularity.^48–50^ Thus, entropy increases with the degree of disorder and is maximum for completely random systems. Accordingly, unexpected fatigue due to sensorimotor disorder in crewmembers may increase the entropy of limb-motion-related signals (acceleration) from sensors.

#### Code availability

Our custom MATLAB codes for all data processing during the current study are not publicly available due to privacy laws and other restrictions but are available from the corresponding author on reasonable request.

### Statistical analysis

We used SPSS (version 24, Chicago, Il, USA) to complete the statistical analysis. For each test (Tandem Walk, Prone-to-Stand, SNOW), mean and one SD values of the dependent variables were calculated for both the EO and EC conditions. The Pearson correlation coefficient (*r*) between the duration of time crewmembers were underwater (or control subjects on land) and all variables were calculated to evaluate the relationship between the subjects’ performance and the time spent in the Aquarius habitat. In cases where the relations were significant, repeated measures ANOVA assessed the effects on performance. The level of statistical significance was set at *p* ≤ 0.05. In cases where the ANOVA provided a statistically significant effect, post hoc analyses (Least Significant Difference) were used to distinguish between the four repeated measures. The dependent variables for the tandem walk test were: Displacement Area of Head, Trunk and Pelvis, and right and left Gait Regularity Mean, SD, and Range. The dependent variables for Prone-to-Stand test were: total sway area and maximum ML and AP sway excursion. The dependent variables for SNOW test were: jerk during horizontal and vertical movements, and sample entropy of horizontal and vertical motions. All SNOW variables were assessed for performance with the legs in the air and on the ground.

### Data availability

The datasets generated during and/or analyzed during the current study are not publicly available due to privacy laws and other restrictions but are available from the corresponding author on reasonable request.
